# Early-Stage Estimated Value of Blend Sign on the Prognosis of Patients with Intracerebral Hemorrhage

**DOI:** 10.1155/2018/4509873

**Published:** 2018-05-06

**Authors:** Weijun Wang, Ningquan Zhou, Chao Wang

**Affiliations:** Department of Neurosurgery, Qiannan People's Hospital, Guizhou 558000, China

## Abstract

**Background and Purpose:**

This study aimed to explore the relationship between blend sign and prognosis of patients with intracerebral hemorrhage (ICH).

**Methods:**

Between January 2014 and December 2016, the results of cranial computed tomography imaging within 24 h after the onset of symptoms from 275 patients with ICH were retrospectively analyzed. The patients with or without blend sign were compared to observe and analyze the difference in coagulation function abnormality, rebleeding, mortality, and bad prognosis rates in the early stages.

**Results:**

Of the 275 patients with ICH, 47 patients had Blend Sign I (17.09%) and 17 patients had Blend Sign II (6.18%). The coagulation function abnormality rate had no statistical difference among Blend Sign I, Blend Sign II, and conventional groups (*P* > 0.05). In the Blend Sign I group, the rebleeding rate was 4.26%, bad prognosis rate was 25.53%, and mortality rate was 6.38%, which were not statistically significantly different compared with those in the conventional group (*P* > 0.05). The rebleeding rate in the Blend Sign II group was 47.06%, bad prognosis rate was 82.35%, and mortality rate was 47.06%, which were statistically significantly different compared with those in the conventional and Blend Sign I groups (*P* < 0.05).

**Conclusions:**

For the patients associated with Blend Sign I, the prognosis was equivalent to that in the conventional group, with no statistically significant difference. The rebleeding, bad prognosis, and mortality rates were higher in the Blend Sign II group than in the conventional group and deserved more attention.

## 1. Introduction

**Figure 1:**
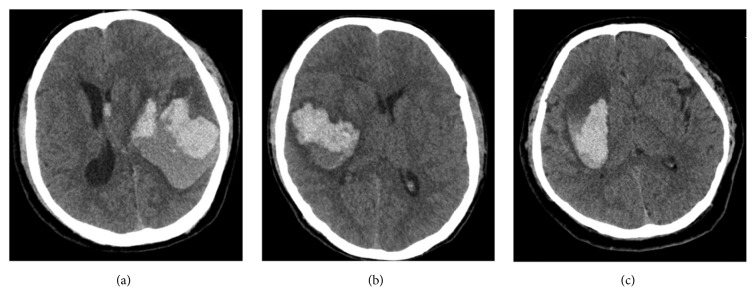
Classic cranial CT scans of Blend Sign I. The whole hematoma was composed of low-density and high-density hematomas with a clear boundary, The difference in the CT measurement value was higher than 18 HU.

**Figure 2:**
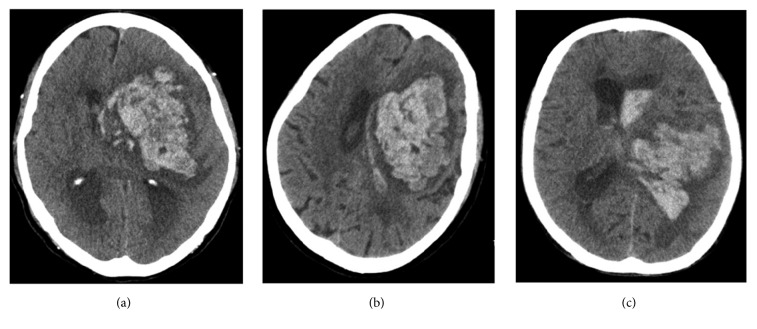
Classic cranial CT scans of Blend Sign II. The whole hematoma was composed of low-density and high-density hematomas without a clear boundary (crossing each other). The difference in the CT measurement value was higher than 10 HU.

Intracerebral hemorrhage (ICH) is a common cerebrovascular disease with high mortality and disability rates [1–3], accounting for about 10%–30% of all strokes [4]. Several factors influence prognosis. The commonly used evaluation methods include Glasgow Coma Scale (GCS) score during hospitalization, gender, coagulation function, and volume of hematoma [5–7]. The lower GCS score, older age, more abnormal coagulation function, and larger bleeding volume easily lead to poor prognosis. However, in clinic, even the combination of various evaluation indexes cannot accurately predict patient prognosis. In particular, at the early stage of disease onset, the prognosis is hard to predict. Li et al. [8] first proposed a blend sign phenomenon based on the imaging morphology of hematoma in patients with ICH. The hematoma in the acute stage comprised two hematomas with different densities, as revealed by cranial computed tomography (CT). The boundary between the hematomas was clear and easily distinguished with naked eyes. The difference in the CT measurement value between the two components with different densities was at least 18 HU. Furthermore, Li et al. proposed that this phenomenon was related to secondary neurologic deterioration. Such imaging signs were also observed in the present study. Besides, another blend sign that was different from the aforementioned phenomenon was noted. It was found in the cranial CT scans of patients with acute ICH. The hematoma in the acute stage still comprised hematomas with two densities, which could be easily distinguished with naked eyes. However, no clear boundary existed between them, and they were distributed in a high-low-density pattern (Figure 2). The blend signs proposed by Li et al. and those in the present study were named as Blend Sign I (Figure 1) and Blend Sign II (Figure 2), respectively. Some of the challenges that needed to be addressed were as follows: detecting a relationship between the blend sign and prognosis, if any; identifying the components of low-density hematoma; and exploring whether the component with density lower than that of acute hematoma was possibly cerebrospinal fluid, fresh blood, or a mixture of these. The present study explored the significance of the two blend signs, the evaluation on prognosis, and the possible mechanism of occurrence of Blend Sign.

## 2. Methods

### 2.1. Patients

A total of 275 patients with ICH were admitted and cured in the Neurosurgery Department, the People's Hospital of Qiannan, from January 2014 to December 2016. Patients with hematoma morphology Blend Sign I on the cranial CT scan were placed in the Blend Sign I group, those with hematoma morphology Blend Sign II in the Blend Sign II group, and those without blend sign in the conventional group. Subsequently, the incidence, rebleeding, mortality, and bad prognosis rates at the time of discharge among the three groups were compared and analyzed. Low-density hematomas from two patients were selected to perform biochemical detection and were compared with the components in the blood.

### 2.2. Inclusion Criteria

The inclusion criteria were as follows: patients with spontaneous ICH confirmed by 64-row cranial CT; patients who received cranial CT examination within 24 h of disease onset and had no diagnosed hemorrhagic disorders or severe underlying diseases; and patients whose family did not give up therapy.

### 2.3. Exclusion Criteria

The exclusion criteria were as follows: patients with causes of hemorrhage including cerebrovascular malformation, arterial aneurysm, tumor bleeding, cerebral trauma, and bleeding tendency such as hemophilia and disseminated intravascular coagulation; patients with intraventricular hemorrhage and spontaneous subarachnoid hemorrhage; patients who received cranial CT examination after 24 h of disease onset and had severe underlying disease before ICH; and patients whose family gave up therapy.

### 2.4. Definition of Blend Sign I and Blend Sign II

Definition of Blend Sign I [6] is as follows: (1) there is blending of relatively hypoattenuating area with adjacent hyperattenuating region within a hematoma; (2) there is not a well-defined margin between the hypoattenuating area and adjacent hyperattenuating region that is easily recognized by the naked eye; (3) the hematoma should have at least an 18-Hounsfield-unit difference between the 2 density regions; (4) the relatively hypoattenuating area was not encapsulated by the hyperattenuating region.

Definition of Blend Sign II is as follows: (1) cranial CT was performed within 24 hours of onset; (2) the hematoma is made up of two kinds of hematoma of different density which can be recognized by the naked eye; (3) the boundary is blurred between the 2 density hematomas; (4) the CT value of two kinds of hematoma is at least 10-Hounsfield-unit difference (CT setting: window width: 120, window level: 40).

### 2.5. Cranial CT Imaging Results

The results of patients' cranial CT imaging were obtained from the imaging department and evaluated by the author. This study was approved by the ethics committee of the hospital.

### 2.6. Instrument, Reagent, and Method of Low-Density Hematoma Biochemical Test and Blood Index Test

The instrument used is ABBOTT ARCHITECT c16000 automatic biochemistry analyzer.

Reagent and method are as follows: Creatine Kinase Kit (Creatinine Phosphate Method); Aspartate Aminotransferase Kit (Aspartate Substrate Method); ICT Reference Solution (Direct Potentiometric Method).

### 2.7. Prognosis Evaluation Indexes

The rebleeding, bad prognosis [Glasgow Outcome Score (GOS) ≤ 3], and mortality rates of patients at the time of discharge from the three groups were compared. GOS is a 5-level score: (1) dead; (2) vegetative state (meaning the patient is unresponsive, but alive; a “vegetable” in lay language); (3) severely disabled (conscious but the patient requires others for daily support due to disability); (4) moderately disabled (the patient is independent but disabled); (5) good recovery (the patient has resumed most normal activities but may have minor residual problems).

### 2.8. Statistical Analysis

SPSS17.0 software (SPSS, IL, USA) was used to analyze the data. Measurement data were expressed as mean ± standard deviation and analyzed using the *F* test. Enumeration data were analyzed using the* χ*^*2*^ test, the prognostic factors analyzed the univariate and multivariate logistic regression, and the inspection level was *α* = 0.05. A *P* value less than 0.05 was termed as statistically significant.

## 3. Results

### 3.1. Study Participants

A total of 275 patients with ICH were enrolled, including 185 males and 90 females with age range of 30–93 years and average age of 59.59 ± 12.53 years (Table 1). The study included 246 patients with cerebral hemisphere hemorrhage, 18 with cerebellar hemorrhage, and 11 with brainstem hemorrhage. Further, 64 patients were associated with blend signs (63 with cerebral hemisphere hemorrhage, 1 with cerebellar hemorrhage, and none with brainstem hemorrhage). Of these, 47 patients were associated with Blend Sign I and 17 with Blend Sign II. A total of 211 patients were in the conventional group. No statistically significant differences were observed in gender, age, systolic pressure, hematoma volume, GCS score at admission, and coagulation function abnormity rate among the three groups (*P* > 0.05) (Table 1).

### 3.2. Comparison between Low-Density Hematoma Biochemical Test and Blood Index Test

**Figure 3:**
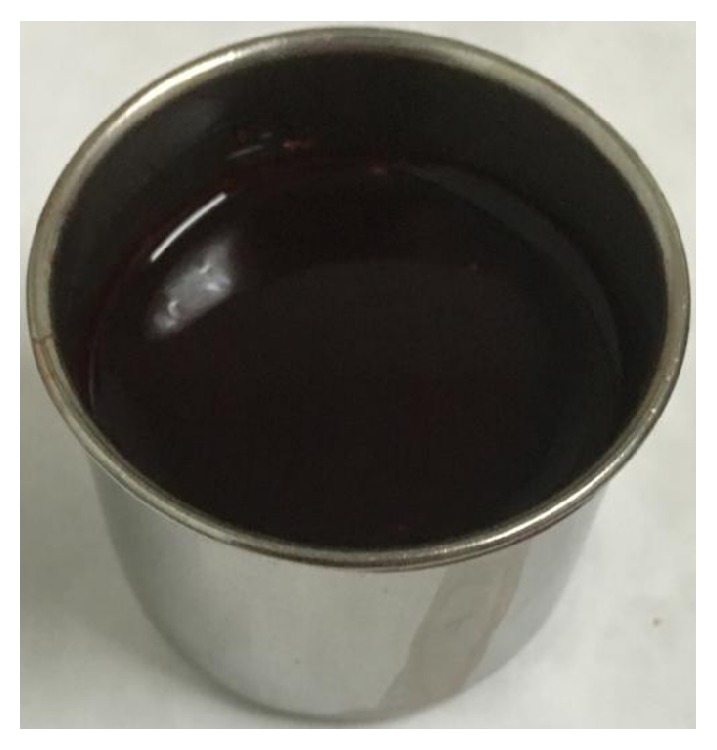
Low-density hematoma was dark red blood clot observed with naked eyes; it was slightly viscous and easily pumped during surgery.

The biochemical test results and blood index text results of the low-density hematoma were compared in the Blend Sign I group. The observations are shown in Table 2. Low-density hematoma was dark red blood clot (Figure 3).

Reference ranges of blood are as follows: chlorine 98–108 mmol/L, creatine kinase 22–269 U/L, and aspartate aminotransferase 0–45 U/L.

Reference ranges of cerebrospinal fluid are as follows: chlorine 119–108 mmol/L, creatine kinase 0–15 U/L, and aspartate aminotransferase 0–15 U/L.

### 3.3. Classic Cranial CT Scans of Blend Sign I and Blend Sign II

The classic CT scan of Blend Sign I and Blend Sign II is shown in Figures 1 and 2, respectively.

### 3.4. Prognosis Evaluation

The comparisons of rebleeding, mortality, and bad prognosis rates among the three groups are shown in Table 3. Comparison of absorption time of hematoma and the drainage time of hematoma with minimally invasive operation from three groups is shown in Table 4.

## 4. Discussion

Most studies have evaluated the prognosis of patients with ICH using factors such as age, hematoma volume, and GCS score at admission [5–7], but the prognosis is still hard to predict. Predicting prognosis is even harder especially at the early stage of onset. Recently, some researchers used the spot sign to predict the risk of hematoma enlargement [9, 10]. However, this method has not been widely used due to the poor condition of CT angiography application in some hospitals. The present study explored the preliminary evaluation of the prognosis via early-stage imaging using the relationship between patient prognosis and blend sign.

**Figure 4:**
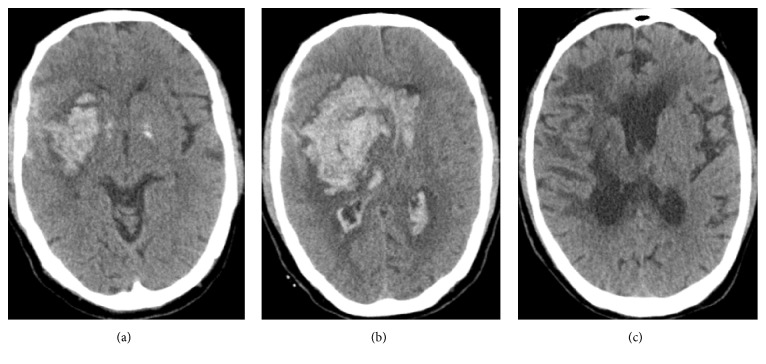
Cranial CT scan of patients with ICH showed Blend Sign II. (a) Hematoma volume is about 20 ml on admission. (b) Cranial CT scan shows that volume of hematoma was 83 ml on 1 hour after admission. (c) GOS of patient was 2 at discharge.

Of the 275 patients with ICH, 47 had Blend Sign I (16.85%), consistent with the findings of Li et al. (16.9%) [8] and Sporns et al. (20.3% 37/182) [11]. However, only 17 patients had Blend Sign II (6.09%). Moreover, no significant difference was observed in gender, age, systolic pressure, hematoma volume, GCS score at admission, and coagulation function abnormality rate among the three groups (*P* > 0.05) (Table 1). Whether the low-density hematoma was related to coagulation function abnormality was doubtful before investigation. No statistically significant difference was found in the coagulation function abnormality rate between the patients with or without blend signs (Table 1). The mechanism underlying the development of unique morphological imaging manifestations of hematoma was unclear, especially for Blend Sign I, and the CT value and cerebrospinal fluid concentration were slightly high. All the patients underwent cranial CT examination within 24 h after the onset of ICH to exclude the possibility of low-density hematoma. Meanwhile, the pressure inside the hematoma in the acute stage was high, and hence the possibility of cerebrospinal fluid entering into the hematoma cavity was low. Besides, most of the ICH did not enter into the cavum subarachnoid and ventricular system. The cerebrospinal fluid did not have a pathway and hence could not enter into the hematoma cavity. Thus, low-density hematoma might not be cerebrospinal fluid. Low-density hematomas from two patients in the Blend Sign I group were selected for this study. Low-density hematoma was a slightly viscous dark red nonclotting blood, which could be observed with naked eyes (Figure 3); it was also easily drained during surgery. The patients with small hematoma volume in Blend Sign I group received nonsurgical treatment. Because of the presence of low-density hematoma, the absorption time of hematoma in Blend Sign I group was shorter than that in conventional group and Blend Sign II group. In patients with large hematoma volume and minimally invasive operation in Blend Sign I group, the drainage time of hematoma was also shorter than that of conventional group and Blend Sign II group (Table 4). Low-density hematoma from two patients with Blend Sign I was selected to perform biochemical detection and was further compared with patients' peripheral blood. The results indicated that chloride ion concentrations in low-density hematoma from the two patients were slightly lower than those in the blood (Table 2). It was assumed that low-density hematoma was composed of cerebrospinal fluid and some blood. The chloride ion concentration in low-density hematoma was higher than that in the peripheral blood because the concentration in the cerebrospinal fluid was higher than that in the blood. Since low-density hematoma was composed of serum and part of blood and the concentrations of creatine kinase and aspartate aminotransferase in hematoma were close to those in the peripheral blood, low-density hematoma was presumed to comprise mainly blood. The low-density hematoma component of Blend Sign I was serum precipitated from blood clot mixed with blood. The analysis of the patients from the three groups indicated that rebleeding, bad prognosis, and mortality rates in the Blend Sign I and Blend Sign II groups were different; the prognosis in Blend Sign I and conventional groups was similar (Table 3). However, the rebleeding rate in the Blend Sign II group was up to 47.06% (8/17), which was much higher than that in the conventional (10.43%) and Blend Sign I groups (4.26%) (*P* < 0.001). The possible reason was that the mechanism of hematoma formation was active bleeding, and low-density hematoma was new blood. The new and old hematomas mixed and formed the imaging morphology of Blend Sign II. This led to a continuous increase in the volume of hematomas, influencing patient prognosis (Figure 4). The bad prognosis rate was up to 82.35% (14/17), and the mortality rate was 47.06% (8/17), which were significantly higher than those in the Blend Sign I and conventional groups (Table 3). The prognosis of Blend Sign II was poor.

We have statistically analysed blend sign and the common prognostic factors (age, sex, hematoma volume, rebleeding, and GCS) of prognosis of patients with ICH. Univariate analysis showed that Blend Sign II age, GCS score, and rebleeding were closely related to the poor prognosis (*P* < 0.05) (Table 5). Multivariate analysis was found to be OR = 4.792  (OR > 1) in Blend Sign II, 95% CI: 1.065–21.568, *P* = 0.041 (*P* < 0.05) (Table 6). Blend Sign II age and hemorrhage were independent risk factors for predicting poor prognosis of ICH. In this study, the reported sensitivity, specificity, positive and negative predictive values of Blend Sign II for predicting bad prognosis were 13.08%, 98.21%, 82.35%, and 63.40%.

Many factors influence the prognosis of patients with ICH. At the early stage, the prognosis is hard to evaluate. If the patients with ICH show a Blend Sign II at the early stage, most of them have bad prognosis, which should be alerted. However, because the occurrence rate of Blend Sign II is low [only 17 cases (6.09%) in the present study], more clinical cases are needed to verify the conclusions.

## 5. Conclusions

(1) Patients with Blend Sign I ICH and conventional ICH have an equivalent prognosis.

(2) Patients with ICH showing Blend Sign II in early-stage cranial CT have an extremely bad prognosis, which is helpful in predicting prognosis at an early stage.

(3) The low-density hematoma component of Blend Sign I may be serum precipitated from blood clot mixed with blood.

## Figures and Tables

**Table 1:** Comparison of general information of the patients from the three groups at admission.

	Conventional group (*n* = 211)	Blend Sign I group (*n* = 47)	Blend Sign II group (*n* = 17)	*F*/*χ*^2^	*P*
Gender (male%)	140 (66.35)	35 (74.47)	10 (58.82)	1.740	0.419
Age (year)	60.86 ± 12.63	53.72 ± 11.50	59.47 ± 9.86	0.942	0.593
GCS (score)	10.30 ± 3.60	11.30 ± 2.66	9.47 ± 4.12	1.095	0.364
Coagulation function abnormity rate (%)	103 (48.82)	31 (65.96)	11 (64.71)	5.570	0.062
Systolic pressure (mmHg)	172.63 ± 30.29	168.59 ± 25.36	178.47 ± 28.28	0.841	0.435
Hematoma volume (ml)	59.11 ± 23.15	49.00 ± 23.17	52.63 ± 29.48	1.041	0.358

**Table 2:** Comparison between low-density hematoma biochemical test and blood test results in the patients.

	Chlorine (mmol/L)	Creatine kinase (U/L)	Aspartate aminotransferase (U/L)
Hematoma (Case 1)	92	1969	353
Blood (Case 1)	98	1082	272
Hematoma (Case 2)	76	406	93
Blood (Case 2)	100	426	68

**Table 3:** Comparisons of rebleeding, mortality, and bad prognosis rates among the three groups.

	Incidence rate (%)	Rebleeding rate (%)	Bad prognosis rate (%)	Mortality rate (%)
Conventional group	211 (77.06)	22 (10.43)	81 (38.38)	32 (15.17)
Blend Sign I group	47 (16.85)	2 (4.26)	12 (25.53)	3 (6.38)
Blend Sign II group	17 (6.09)	8 (47.06)	14 (82.35)	8 (47.06)
*χ*_1_^2^	196.358	0.350	2.756	1.835
*χ*_2_^2^	281.952	31.803	10.768	11.062
*χ*_3_^2^	15.884	16.781	16.710	11.795
*P*1	<0.001	0.554	0.096	0.176
*P*2	<0.001	<0.001	0.001	<0.001
*P*3	<0.001	<0.001	0.001	<0.001

*χ*_1_^2^: comparison between conventional and Blend Sign I groups; *χ*_2_^2^: comparison between conventional and Blend Sign II groups; *χ*_3_^2^: comparison between Blend Sign I and Blend Sign II groups.

**Table 4:** Exclusion of craniotomy: conventional group (*n* = 9), Blend Sign I group (*n* = 5), and Blend Sign II group (*n* = 2). Comparison of absorption time of hematoma and the drainage time of hematoma with minimally invasive operation from three groups.

	Nonoperative treatment (%)	Hematoma absorption time (d)	Minimally invasive surgery rate (%)	Hematoma drainage time (d)
Conventional group (202)	77 (38.11)	22.91 ± 5.14	125 (61.88)	6.25 ± 0.81
Blend Sign I group (42)	16 (38.09)	15.58 ± 3.87	26 (61.90)	4.00 ± 0.96
Blend Sign II group (15)	4 (26.66)	22.33 ± 2.46	11 (73.33)	5.66 ± 0.88
*χ*_1_^2^/*t*	0. 00	−3.94	0.00	−5.82
*χ*_2_^2^/*t*	0.783	−0.35	0.783	−4.54
*χ*_3_^2^/*t*	0.634	5.63	0.634	1.54
*P*1	0.997	<0.001	0.997	<0.001
*P*2	0.543	0.363	0.543	0.001
*P*3	0.630	<0.001	0.630	0.068

*χ*_1_^2^/*t*: comparison between conventional and Blend Sign I groups; *χ*_2_^2^/*t*: comparison between conventional and Blend Sign II groups; *χ*_3_^2^/*t*: comparison between Blend Sign I and Blend Sign II groups.

**Table 5:** Univariate analysis of predictors for early prognosis.

Variable	OR	95% CI	*P*
Gender (male%)	1.825	0.921–3.618	0.085
Age (year)	1.059	1.030–1.089	<0.001
GCS (score)	0.686	0.612–0.769	<0.001
Hematoma volume (ml)	1.006	0.993–1.018	0.362
Blend Sign I	1.032	0.419–2.541	0.945
Blend Sign II	4.894	1.094–21.893	0.038
Rebleeding	14.341	4.806–42.800	<0.001

**Table 6:** Multivariate analysis of predictors for early prognosis.

Variable	OR	95% CI	*P*
Age (year)	1.056	1.028–1.085	<0.001
GCS (score)	0.686	0.618–0.761	<0.001
Blend Sign II	4.792	1.065–21.568	0.041
Rebleeding	14.871	5.064–43.668	<0.001
